# What Makes for a Positive Patient Experience in Chronic Pain Care? A Qualitative Evaluation of Factors Affecting Veteran Patient Satisfaction Across Two Individualized Pain Care Interventions in VA Primary Care

**DOI:** 10.1007/s11606-025-09630-3

**Published:** 2025-05-27

**Authors:** Natalie Purcell, Beth M. DeRonne, Hope A. Salameh, Erin E. Krebs, Karen H. Seal, William C. Becker, Hildi J. Hagedorn

**Affiliations:** 1 Center for Data to Discovery and Delivery Innovation, San Francisco VA Health Care System, San Francisco, CA USA; 2 Department of Social and Behavioral Sciences, University of California, San Francisco, CA USA; 3 Center for Care Delivery and Outcomes Research, Minneapolis VA Health Care System, Minneapolis, MN USA; 4 Division of General Internal Medicine, University of Minnesota Medical School, Minneapolis, MN USA; 5 Departments of Medicine and Psychiatry, University of California, San Francisco, CA USA; 6 Pain Research, Informatics, Multimorbidities and Education (PRIME) Center, VA Connecticut Healthcare System, West Haven, CT USA; 7 Department of Internal Medicine, Yale School of Medicine, New Haven, CT USA; 8 Department of Psychiatry & Behavioral Sciences, University of Minnesota Medical School, Minneapolis, MN USA

**Keywords:** chronic pain, patient experience, patient satisfaction, veterans, qualitative methods

## Abstract

**Background:**

Veterans are at high risk for pain-related disability, medication overdose, and opioid-related deaths. In response, Veterans Affairs (VA) healthcare systems are working to implement innovative, multimodal pain care. Recently, the Veterans’ Pain Care Organizational Improvement Comparative Effectiveness (VOICE) study compared two interventions that provide individualized pain care and opioid tapering—an interdisciplinary integrated pain team (IPT) and pharmacist collaborative management (PCM).

**Objective:**

Informed by VOICE qualitative interview data, this paper examines patient experiences with IPT and PCM and identifies factors that affected patient satisfaction across both interventions.

**Approach:**

We conducted qualitative, semi-structured interviews with 63 veteran patients who participated in VOICE. The first set of interviews (*n* = 32) examined patients’ experience with the VOICE interventions and solicited suggestions for improvement. The second set (*n* = 31) examined patients’ experiences with telehealth in VOICE and inquired about changes to pain-care access and delivery associated with the COVID-19 pandemic. We used rapid analysis procedures to identify themes across both sets of interviews.

**Key Results:**

Veterans enrolled in both VOICE interventions described how they learned to better live with and self-manage pain. Across interventions, key factors that facilitated a positive patient experience included the opportunity to develop a long-term relationship with the clinician or care team, meaningful patient involvement in treatment planning and decision-making, adequate variety and accessibility of options for treatment and self-management, and ease of communication and care coordination.

**Conclusion:**

Although IPT and PCM are unique interventions with significant differences from one another, the fundamental factors that influenced patients’ satisfaction were common to both interventions, including the opportunity to develop a therapeutic patient–clinician relationship, engagement in shared decision-making, adequate care access, and support for care coordination. These factors, which are relevant across different pain care interventions and contexts, should be key considerations as healthcare organizations design and implement pain care interventions.

## INTRODUCTION

Chronic pain, or pain that persists after 3 months, affects an estimated three in ten US military veterans.^1^ As a result, veterans are at high risk for pain-related disability, medication overdose, and opioid-related deaths.^2–5^ As the limited benefits and potential harms of long-term opioid therapy have come into focus,^6–10^ the Department of Veterans Affairs (VA) and other healthcare organizations have recognized a need to develop innovative, multimodal approaches to chronic pain care,^11^ especially those that can be implemented widely and integrated into primary care settings.

In response, the Veterans’ Pain Care Organizational Improvement Comparative Effectiveness (VOICE) study^12^ evaluated two promising care models for improving pain and reducing opioid use in VA primary care. VA patients with chronic pain on long-term opioid therapy were randomized to receive either an integrated pain team (IPT) or pharmacist collaborative management (PCM) intervention for 12 months. In IPT, an interdisciplinary clinical team provided pain-care planning with a focus on multimodal non-pharmacological therapies plus regular behavioral-activation sessions. In PCM, a clinical pharmacist provided pain-care management, including medication optimization with monitoring of symptoms and progress. Clinicians in both interventions facilitated referrals to ancillary pain services and provided education in pain self-management as indicated. Notably, the VOICE trial found that both IPT and PCM resulted in modest improvements in pain and substantial reductions in opioid dosage, with no significant difference in effectiveness between interventions.^12^

An additional aim of the VOICE trial—the focus of this paper—was to examine patients’ experiences with the VOICE study interventions through semi-structured interviews. Evaluating patient experiences with pain care interventions is important: to date, studies suggest that both patients and clinicians find chronic pain care challenging, frustrating, and demoralizing.^13–15^ Difficult relationships between patients and clinicians are common, and both can feel trapped in power struggles related to pain medication and treatment decisions.^16–21^ Discussions about opioids can be particularly challenging, with patients feeling unheard or labeled as drug seekers and difficult patients.^13,22^ Moreover, even with pharmacological treatment, pain can continue to negatively impact patients’ health, functioning, and quality of life.^23–25^ Although there is evidence that multimodal and integrated care models like IPT and PCM can reduce pain,^12,26^ improve functioning,^12,26–28^ and decrease reliance on opioids,^12,27,28^ it is not yet clear how these interventions impact patients’ care experiences or what factors are important in shaping those experiences.

In the present study, we conducted qualitative semi-structured interviews to examine patients’ experiences with IPT and PCM. Across both interventions, we sought to understand factors that affect patient satisfaction with pain care and to identify lessons that can guide the future design and implementation of evidence-based care models for pain.

## APPROACH

### Ethics Statement

The VA Central Institutional Review Board approved this study, and all participants gave informed consent.

### Study Design

We conducted two sets of semi-structured interviews with patients (*n* = 63) who completed VOICE participation to better understand their experiences with the study interventions and to identify strategies to improve the interventions. The first set of interviews (*n* = 32) focused on patients’ overall VOICE experience and suggestions for improvement. The second set of interviews (*n* = 31), added to the study design as a result of the COVID-19 pandemic, examined patients’ experiences with telehealth in VOICE and inquired about changes to pain-care access and delivery related to the COVID-19 pandemic.

### Setting

VOICE study sites included a geographically diverse group of ten VA medical centers serving the Northeastern, Southern, Midwestern, and Western United States. In addition to providing the VOICE interventions, all sites offered standard pain services (including physical therapy) and VA-approved complementary and integrative health (CIH) services for pain (e.g., yoga, acupuncture, chiropractic care). Some sites offered additional, non-mandated modalities (e.g., mindfulness-based stress reduction). Although VOICE services continued throughout the COVID-19 pandemic, the pandemic negatively affected access to VA pain services^29^ and may have limited VOICE participants’ access to ancillary and CIH services recommended in their VOICE care plans. (When VA sites are unable to provide timely access to mandated services, VA pays for care in the community, but community care access varies and was also impacted by the pandemic.^30^)

### Participants

Participants were recruited from all ten VOICE sites. All participants met study inclusion criteria, reporting pain nearly every day for at least 6 months; a score of at least 5 on the Pain, Enjoyment of Life and General Activity 3-item scale (range 0–10); and opioid analgesics for at least 3 consecutive months (at least 20 morphine milligram equivalents per day). For both interview sets, we used quota-based sampling to ensure inclusion of key subgroups important to our analysis. Specifically, for each interview set, we established a minimum number of participants by study intervention (≥30% per intervention); intervention completion status (≥20% non-completers); opioid dose (≥20% high-dose, defined as 70+ mg/day); gender (≥20% women); race/ethnicity (≥20% non-white racial and ethnic minorities); and age (≥20% under 50 years old). These quotas were established to ensure adequate diversity in the samples through intentional inclusion of potentially under-represented demographic groups.^31^ A summary of the characteristics of all patient interview participants is presented in Table 1.

**Table 1:** Interview Participants

Patient participant characteristics		General experience interviews (*n* = 32)	Pandemic impacts interviews (*n* = 31)
Study intervention			
	IPT	16	19
	PCM	16	12
Intervention completion status			
	IPT completer	13	15
	IPT non-completer	3	4
	PCM completer	12	10
	PCM non-completer	4	2
Opioid dose level			
	Moderate	24	25
	High	8	6
Gender			
	Man	23	19
	Woman	9	12
Race			
	White	20	17
	Racial and ethnic minorities	12	14
Age			
	50 and over	25	23
	Under 50	7	8

### Procedures

A senior implementation scientist (HH) and a qualitative methodologist (NP) with extensive experience in health services research led the team.^32–37^ The team also included two interviewer-analysts who were mentored by the team’s leads in qualitative interviewing and analysis (HS, BD).^38,39^ The team met routinely throughout the study to discuss methodological questions, to foster consistency in interviewing and analysis techniques, and to promote rigor through active conversation, questioning, and consensus-building.

For each set of qualitative interviews, the team developed an original semi-structured interview guide. These were adapted from guides the team leads employed in similar studies,^32–35,37,40–42^ and designed to elicit feedback across pre-identified topics relevant to patient experience. Interview topics and sample questions are presented in Table 2.

**Table 2:** Interview Topics and Sample Questions

**VOICE patient experience interview topics**	**Sample question(s)**
Life & Health Impacts	• How has your life and your health changed since you began working with [the Integrated Pain Team or Pharmacist Pain Care]?
What Worked Well and What Did Not Work Well	• Overall, how well did [the Integrated Pain Team or Pharmacist Pain Care] meet your needs as a patient? • What worked well for you? (Why?) • What didn’t work well for you? (Why?)
Relationship/Communication w/VOICE team	• How would you describe your relationship and communication with your [Integrated Pain Team or Pharmacist Pain Care] clinicians?
Coordination b/t VOICE & Primary Care	• Did any of your regular VA clinicians outside of the VOICE study (for example, your primary care doctor or nurse) talk to you about your participation in the VOICE study? What did they have to say?
Impact on Attitudes toward Health/Healthcare	• How has your VOICE study experience affected your feelings about your health and your healthcare? • Has your VOICE experience affected how you feel about your ability to improve your health? Why or why not? • Has working with your VOICE treatment team affected how you feel about the VA healthcare system? If so, in what ways?
Arm Preference (if applicable)	• Before you were assigned to [the Integrated Pain Team or Pharmacist Pain Care], did you have an opinion about which service you hoped to receive? • [If yes]: Why were you more interested in [the Integrated Pain Team or Pharmacist Pain Care]?
Improvement Opportunities	• Looking back on your overall experience with [the Integrated Pain Team or Pharmacist Pain Care], how could we improve to better serve Veterans like you?
Willingness to Recommend Intervention	• Would you recommend [the Integrated Pain Team or Pharmacist Pain Care] to other Veterans? Why or why not?
**COVID-19 pandemic impacts interview topics**	
VOICE Access During Pandemic	• Was your access to VOICE services been affected by the pandemic in any way? How so?
Pain Care Access During Pandemic	• Has your access to health care or pain care been affected? How so?
Telehealth Experiences	• In general, how have you felt about receiving care through phone or video versus in person? • When it comes to phone and video care, what (if anything) has been challenging for you? What don’t you like about it, and why? • When it comes to phone and video care, what (if anything) has worked well for you? What do you like about it, and why?
Telehealth Relationships and Communication	• How has getting care through phone or video affected your relationships with your care clinicians?
Telehealth Suggestions	• What could your VOICE team or VA do to improve the care you’re receiving by phone or video?
Telehealth Preferences	• If you had a choice to receive your future pain care through phone, video, in-person, or a mix, what would you choose and why?

Interviews were conducted via telephone, lasted approximately 30 to 60 min, and were audio-recorded and transcribed (with the exception of three interviews not recorded due to patient privacy concerns; detailed notes were taken during these three interviews). The first set of interviews was conducted August 2020 through June 2021; the second, July 2021 through April 2022.

Qualitative analysis took place over three phases. First, interviewers completed a free-form, unstructured memo^43^ (typically one to two paragraphs) summarizing central “takeaways” following each interview and describing the interview’s overall tone and tenor. Next, analysts used a template-based rapid analysis technique developed for health services research.^39,44,45^ Rapid analysis was designed to be time- and resource-efficient, yielding results comparable to traditional qualitative methods.^46–48^ We selected rapid analysis methods to facilitate the timely identification of emerging findings for communication to study partners.^49^ Using both audio-recordings and transcripts, a member of the analysis team prepared a structured summary of each interview using a spreadsheet-based template organized by topics drawn from the interview guide (Table 2). The analyst summarized interview content for each topic and populated relevant quotations into the template. To ensure consistency among analysts, a second analyst examined a subset of interviews (20%) from each set, noting additional observations or conflicting interpretations. Routine team meetings provided an opportunity to discuss differences in interpretation and approach and to achieve consistency in rapid analysis techniques.

The third phase of analysis involved the identification and refinement of themes through an iterative, collaborative process.^50^ Team members began by reading all memos and templated summaries and, as needed, returning to audio-recordings, notes, and/or transcripts for clarification and context. The team then populated an Excel-based matrix with preliminary themes for each topic. Alongside each theme, the team listed supporting citations, relevant excerpts from memos/templates, key quotations, case examples, and counterexamples. Theme identification was a collaborative process, with individual team members mapping content to the matrix between team meetings, then working together during team meetings to discuss, organize, and condense content and, ultimately, to describe and refine themes.

## FINDINGS

We present our findings across three broad domains (*Overall Intervention Impact*, *Intervention Factors Affecting Patient Experience*, and *Telehealth Versus In-Person Care Delivery*). Each domain encompasses several inter-related themes, as presented in Table 3.

**Table 3:** Interview Themes by Domain

Domain	Theme title	Short description of theme
Overall intervention impact		
	Collaborative Pain Care Empowers Patients	Pain education and collaborative pain care-planning can foster a sense of patient empowerment and greater engagement in healthcare decision-making.
	Self-Management Can Improve Quality of Life	Learning techniques for self-management of pain can improve patients’ quality of life, whether or not their pain is reduced.
	Reducing Opioids Can Improve Functioning	Collaborative care plans that reduced opioids often improved perceived energy levels, cognitive clarity, and daily functioning.
	Reducing Opioids Can Increase Pain	Opioid reductions could also be difficult and burdensome and result in greater pain for some patients (including some who nonetheless found the reductions beneficial and worthwhile).
	Successful Care Plans Are Comprehensive, Not Just Opioid-Focused	Patients benefited most from comprehensive pain care-planning, not care-planning focused primarily on opioid reduction.
Intervention factors affecting patient experience		
	Patients Value Meaningful Engagement in Care-Planning	Patients wanted to be involved in care-planning, decision-making, and goal-setting in a meaningful way on an ongoing basis.
	Patients Value Having Multiple Treatment Options	Patients appreciate having a variety of treatment and self-management options to choose from and the opportunity to make informed choices.
	Patients Value Individualized Care	Patients wanted their care plans to be tailored to their specific needs and values—not a one-size-fits-all treatment.
	Patients Value a Caring Clinician-Patient Relationship	Patients wanted a warm, caring relationship with the clinicians on their care team.
	Patients Value Continuity of Care Over Time	Patients appreciated having sustained, focused, ongoing personalized attention from the same clinical team over multiple visits.
	Patients Value Responsiveness and Ease of Communication	Patients appreciated having easy access to their care team, a clear point of contact, and consistent responsiveness to questions and concerns.
	Patients Value Care Coordination Support	Patients found it easier to follow through on their care plans when they received support with basic care coordination.
	Poor Access Creates Frustration and Disrupts Care Plans	Patients could follow through on multi-modal care plans only when there was adequate, timely access to recommended services; inability to access recommended/planned services created frustration and disappointment.
Telehealth versus in-person care delivery		
	Patients Value the Ease, Accessibility, and Convenience of Telehealth	Patient described both video and telephone care as more convenient and less burdensome than in-person care.
	Patients Want the Option of In-Person Care When Indicated	Patients felt that some pain care services require a hands-on exam and must be conducted in person (e.g., visits concerning new problems). They wanted a mix of in-person, video, and phone care options.
	Face-to-Face Meetings Can Facilitate Relationship-Building	Seeing a clinician face-to-face can be an important part of developing a care relationship and rapport, especially early in the relationship.
	Mode of Care Delivery Matters Less Than Quality of Communication	Patients felt the mode of care delivery was less important than the rapport, quality of communication, strength of the relationship, and continuity of the relationship between clinician and patient.

### Overall Intervention Impact

Across both study interventions, patients described how they learned to better live with and self-manage pain. “I know the triggers,” explained one veteran, “I know why my body is reacting towards it more, whether it be environmental or mental.” Patients in both study interventions felt that VOICE equipped them with education, tools, and techniques—such as movement, stretching, and pacing—that improved their quality of life even if their pain did not improve. “All of that stuff that… we talked about, I’m still able to use it now,” affirmed one veteran.

Some patients felt newly empowered and equipped to ask questions about their healthcare, to request information from clinicians, and to become more actively involved in addressing their health concerns and developing care plans. The information provided by VOICE clinicians regarding medications was eye-opening for some and resulted in their thinking more critically about their medication regimens and discussing these more actively with their clinicians. Some reported feeling more confident asking questions and getting involved in their healthcare. One veteran explained:

I think it helped me speak out when there’s something that I’m not comfortable with or not too crazy about, usually I don’t want to try to step on the doctor’s toes, and you know, I want to be open, but I think now… it helped me express when there’s something I have a lot of questions about or that I’m uncomfortable with, that I voice those concerns.

Patients often found that, as a result of participating in VOICE, they had more energy and were able to engage in additional tasks like mowing the lawn or walking to the mailbox to get the mail. “I was able to make work calls,” noted one veteran, “I was able to do more, try to get [to] my other things… that were so far behind.” Patients also reported cognitive benefits, such as forgetting fewer things and being able to think more quickly or clearly (“I’m more alert now”), often attributing these improvements to a reduction in their opioid medications. The results could be life-changing: “I’ve kind of taken my life back. I’m a lot more active, I’ve started walking two, three miles a day … Hardest thing I do now is chase my grandchildren.”

Patients were sometimes surprised to find that their pain severity decreased or stayed the same despite a reduction in opioid dose: changes to their medication regimens and the addition of non-pharmacological pain management strategies could result in less pain than their pre-VOICE treatment plans. “As I started feeling better and taking less meds… I was able to get more active, and the more active I got, the better I felt,” shared one veteran. “Before, I was just existing, and it was to the point where every day was worse than the day before,” confided another, “now, I’m *living*.”

At the same time, even gradual opioid dose reductions could be difficult and burdensome. In both study interventions, some patients reported that their pain severity increased, despite the addition of other medications and self-management strategies. Among patients who were dissatisfied with aspects of their VOICE care, this was a primary complaint. For some, the burden was intense, and significantly impacted their mood and quality of life. For others, it was manageable or counter-balanced by the reduction in opioid-related side-effects. One veteran described the trade-off:

As I withdrew from that number of pain pills, I became aware that I had other problems... I didn’t know that I was going to have the pains in my shoulders, I didn’t know that my right hip now and my lower back would start to hurt so much... I wouldn’t feel probably feel… this as much if I had remained on 200-plus pills a month, but the fact of the matter is, it’s better for my cognitive ability and my safety to be on less.

The small number of interviewed patients who were largely dissatisfied with their VOICE care tended to feel that their treatment team focused primarily on opioid reduction rather than developing a comprehensive, effective pain management plan. Representative of this sentiment, one veteran wished that VOICE “could’ve been more in depth instead of just… oh, we’re going to take your meds.” Some of these patients felt a misalignment between their own objectives for their pain care and the opioid-focused objectives of their treatment team. They did not experience their care as patient-centered, and sometimes felt that they had no real choice: “The federal government wants people off opioids,” one veteran said, “And the pharmacist is the tool that gets that handled.”

### Intervention Factors Affecting Patient Experience

Patients felt most satisfied with their VOICE care when they were involved in care-planning, decision-making, and goal-setting in a meaningful way on an ongoing basis. They appreciated being presented with information about different treatment/self-management options and given the opportunity to make choices. Across both study interventions, satisfied patients felt that their concerns and preferences were heard and respected. They appreciated “the listening, the trying to work with me instead of ‘we think this is best.’” Affirmed one veteran, “I always felt that [the VOICE clinician] took my opinions as something of value.” Although not every VOICE participant felt that they experienced patient-centered care and shared decision-making, patients across both interventions frequently cited these as a notable feature of their VOICE experience and as what they valued most. As a result, these patients felt personally respected by their care team: “It’s almost like they catered to me,” observed one veteran, “They made sure I was able to do everything, and made sure I felt good… Everything was always left up to me… They never forced anything… They’d ask me.”

It was important to patients that their VOICE clinicians genuinely care about them and express compassion and interest in their well-being. Beyond praising specific aspects of the interventions, satisfied patients attributed their positive VOICE experience to their clinicians’ personal demeanor and ability to foster a warm, caring relationship. One veteran explained that he was willing to consider significant adjustments to his medication regimen for the first time because he sensed that his VOICE clinician was sincerely concerned about his pain and well-being and would thus be responsive to his needs: “It seemed like she genuinely cared about how I was going to feel, and how life is going to be going forward. I kind of put my doubts away. And [I] really got into what they recommended and what they wanted me to do.” Conversely, patients who did not have a positive experience often cited the personal demeanor or manner of the clinician as a key factor. For example, they described feeling rushed and not cared about.

Patients felt that both interventions provided more sustained, focused, ongoing personalized attention to their pain care than they had experienced before. Patients appreciated check-in calls and follow-up sessions throughout their 12 months in VOICE; this was important to developing a pain-management plan that would work for them in the long term. It also facilitated relationship-building: “The communication part got really good after we got to know each other and settled in,” said one veteran, “and they knew my case better.” Yet some patients desired even more of both—that is, more frequent check-ins or longer-term care: “It’s always good to have somebody else to talk to when you’re dealing with some of the issues I’m dealing with.”

Satisfied patients praised their VOICE teams’ accessibility and responsiveness. They appreciated knowing that their calls and messages would be returned and having the name of a specific person to reach out to rather than having to leave a voicemail on a generic clinic line. Patients contrasted their VOICE experience to their usual VA experience, where the care system could be difficult to navigate with mixed responsiveness to calls and secure messages. In particular, patients found it helpful to have a single point of contact for queries and questions (often the local VOICE study coordinator): “It’s just helpful to know that somebody is going to call and immediately react to your concerns.”

Patients also appreciated it when their VOICE care team became actively involved in care coordination. Once enrolled in VOICE, they generally found it easier to get medication renewed, to follow through on referrals, and to get support in working through obstacles to care access: “Once everything was in place [in VOICE], you know, gosh, that was a lot easier to get things done.” Patients identified care coordination support as one of the most valuable elements of their VOICE experience and frequently contrasted this with their usual care experience. Some could “not say enough about how helpful [it was] having that knowledge that somebody that can do something is going to be calling.”

Nonetheless, some patients described challenges in following up on their care plans, especially at the height of the COVID-19 pandemic. Sometimes, the services they were referred to required long waits, were only available through care in the community (outside VA), or were simply unavailable for extended periods. When the medical center in which the VOICE team was embedded had challenges related to care access and coordination (common during the pandemic), those affected patients’ VOICE experiences too. For example, one veteran described how “acupuncture was working real good.... the doctor was trying to get me in every two weeks. But because of the long wait lines at the VA at the time I could get in every six weeks, which he says is counter-productive because, you know, I needed it more frequently than that.”

### Telehealth Versus In-person Care Delivery

VOICE services were designed to be telehealth-friendly, with face-to-face modalities (in-person or video) recommended for initial visits and as indicated. For IPT medical clinician visits, face-to-face visits were strongly encouraged. However, as a result of the pandemic, many patients received care exclusively via video or telephone. Interviewed patients saw the VOICE interventions as well-suited to telehealth delivery, and they described both video and telephone care as more convenient and less burdensome than in person care. Reasons cited included the need for travel to in-person care, parking, logistical barriers, and the need to take time off from work. Other patients, especially women, noted that in-person healthcare environments with crowded waiting rooms can be stressful. Some felt more calm, “collected,” “comfortable,” and “at ease” when meeting clinicians from their home. Telehealth allowed some to access services that they would not have otherwise accessed.

Still, patients felt that some pain-care needs—for example, new problems—require a hands-on, in-person exam. Overwhelmingly, they wanted a mix of in-person, video, and phone options. As one veteran put it, “I would say I love the flexibility of making an appointment using video, but if… someone had to examine a body part or like, physically see something … you definitely need a mix.” Some patients also felt that face-to-face interaction, whether by video or in-person, was an important part of developing a relationship and rapport at the start of the care relationship: “I think maybe getting the opportunity to meet the individual that you’ll be working with is always, in my mind, helpful because I know who I’m working with. The remainder of them being over the phone would be fine.”

A strong latent theme across interviews was that the mode of care delivery was less important than the rapport, quality of communication, and strength of the relationship between clinician and patient, as well as the continuity of that relationship over time. No matter how care was delivered, patients wanted the opportunity to build a meaningful, lasting personal connection with their clinician(s): “I think things just need to be more personal, regardless of if it’s video… Even if it is a video conference, it’s best I think if you have some continuity… with the same physician over the course of some time.”

## DISCUSSION

**Figure 1:**
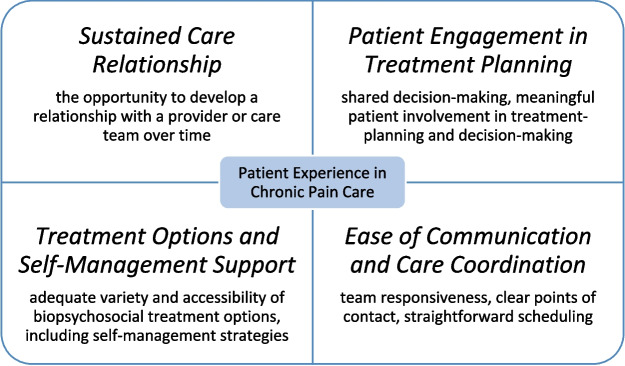
Cornerstones of patient satisfaction in VOICE chronic pain care.

This qualitative evaluation of patient experiences with VOICE’s IPT and PCM interventions identified several factors that impact patients’ experience and should be considered in designing and implementing pain-care interventions. Among interviewed patients, key factors that facilitated a positive, satisfying care experience included the opportunity to develop a long-term relationship with their clinician or care team, meaningful patient involvement in treatment planning and decision-making, accessibility of treatment and self-management options, and ease of communication and care coordination (Fig. 1). Although IPT and PCM are unique interventions with notable differences in design and delivery, the fundamental factors that influenced patients’ satisfaction were common to both interventions and are relevant across many different types of pain interventions.

Above all, patients wanted the opportunity to develop a relationship with their pain-care clinician(s). A key aspect of the VOICE interventions that made this possible was the number and frequency of visits, as well as reasonable continuity in clinicians over time. Patients averaged 11.6 IPT visits (4.3 medical and 5.5 mental-health clinician visits) and 9.9 PCM visits (9.4 pharmacist visits). Generally, patients were assigned consistent IPT and PCM clinicians for 12 months. The care offered in VOICE was almost certainly more resource-intensive than usual primary care–based pain care. But, for many patients, this is what made the difference. They were able to overcome fears about opioid reduction and make significant changes to their care plans because these changes could happen within the context of a supportive, ongoing care relationship.

Patients also saw clinicians’ caring and kindness as essential to developing a positive care relationship, and they valued warmth, empathy, and good listening. Although patients attributed these characteristics to clinicians’ personalities, studies suggest that these are skills that clinicians can intentionally cultivate. For example, Zulman and colleagues^51^ identified several promising strategies to promote “presence and connection with patients in the clinical encounter.” Among them were “listen[ing] intently and completely” and learning “what matters most” to the patient so the patient’s priorities can guide the visit. Zulman and colleagues also emphasized the importance of recognizing and validating emotional cues and “connect[ing]with the patient’s story.” These recommendations are consistent with our findings and may be especially important in the potentially fraught spaces of pain care and opioid tapering: satisfied VOICE patients were those who felt their clinicians listened well, understood what mattered to them, and showed a genuine interest in their needs, preferences, and overall well-being.

Meaningful patient engagement in goal-setting and treatment-planning was fundamental to VOICE patient satisfaction. Patients praised the approach to shared decision-making that they experienced in both VOICE study interventions. Our team’s earlier research on integrated pain teams^37^ also showed a connection between patient satisfaction and perceived control in treatment-planning. Those who felt their own goals drove treatment-planning tended to have positive experiences and reported making more significant changes to their pain care plan. In both that study and this one, patients who saw tapering opioids as the only or primary goal of treatment felt less in control, less invested in their care, and less satisfied. Involving patients in both defining treatment goals and creating care plans was key.

Here, there is considerable alignment between our findings and those of Kim, Rendon, and Starkweather,^52^ who identified multiple processes important to patient-centered chronic pain management in a qualitative study of patients and clinicians. These processes included developing a relationship; understanding the patient’s preferences, needs, and values; partnering to set expectations and negotiate treatment plans; and reassessing after evaluating outcomes and experiences. Like us, they found that it is important to continually engage patients, seeking their input throughout the continuum of pain care.

In VOICE, engaging patients in their pain care involved educating and equipping them with tools for self-management. Satisfied VOICE patients were those who felt empowered to do more for their own health and well-being; they took pride in practicing new skills that they learned through VOICE. It is possible that active self-management may happen more readily among patients who are engaged in shared decision-making and treatment planning. In a recent evaluation of a tailored coaching program for Black veterans with chronic pain, Matthias and colleagues^53^ found that veterans gained confidence in pain self-management and a greater sense of ownership over their pain care by learning to ask effective questions, communicate concerns, express treatment preferences, and share personal goals and values in pain care visits. Additional research is needed, but supporting patients’ active participation and engagement in healthcare visits may facilitate improved engagement in pain self-management strategies.

Self-management, however, is only part of the picture. Access to a variety of treatment options, and the opportunity to develop and follow through on a tailored care plan, was key to VOICE patient satisfaction. Patients valued access to both pharmacological and non-pharmacologic modalities, including acupuncture, chiropractic care, and exercise therapy. It was important to them that recommended options be relatively easy to access. This was not always the case in VOICE, especially during the pandemic. Delayed access to recommended modalities was a significant source of patient frustration and could leave patients feeling disempowered and unable to follow through on their care plans. Prior research by members of our team also suggests that access can be a key issue in multimodal pain care.^37^ Pain teams may wish to mitigate access issues by developing clear referral pathways to recommended modalities and working with ancillary service providers to facilitate patient access and care coordination.

Overall, ease of communication, coordination, and scheduling were important factors shaping patients’ experience. When VOICE teams played an active role in coordinating patient care and supporting patients in accessing ancillary services, patients were impressed and grateful. They also felt that this made it easier for them to follow through on their care plans. Patients who were satisfied with VOICE emphasized the accessibility of their clinician(s) and their responsiveness between visits. Here, the VOICE study coordinators undoubtedly played an important role, serving as points of contact and relaying messages. Replicating the coordinator role in clinical settings could be challenging but may yield a significant benefit if feasible.

As the VA and other healthcare organizations move to expand access to integrated, multimodal chronic pain care, it is important to focus on fundamental factors that can affect the patient experience across different interventions and contexts. Attentiveness to the underlying organizational infrastructure and how it supports patient access and care coordination is critical. So too is equipping clinicians to deliver personalized, person-centered, relationship-based care in collaboration with the patients they serve.

## References

[1] **Qureshi AR, Patel M, Neumark S, et al.** Prevalence of chronic non-cancer pain among military veterans: a systematic review and meta-analysis of observational studies. BMJ Mil Health. Published online December 12, 2023:e002554. 10.1136/military-2023-002554 10.1136/military-2023-002554 38124087

[2] **Bennett AS, Watford JA, Elliott L, Wolfson-Stofko B, Guarino H.** Military veterans’ overdose risk behavior: Demographic and biopsychosocial influences. Addict Behav. 2019;99:106036. 10.1016/j.addbeh.2019.106036 31494452 10.1016/j.addbeh.2019.106036 PMC6791780

[3] **Bennett AS, Guarino H, Britton PC, et al.** U.S. Military veterans and the opioid overdose crisis: a review of risk factors and prevention efforts. Ann Med. 2022;54(1):1826-1838. 10.1080/07853890.2022.2092896 35792749 10.1080/07853890.2022.2092896 PMC9262363

[4] **Peltzman T, Ravindran C, Schoen PM, et al.** Brief Report: Opioid-Involved Overdose Mortality in United States Veterans. Am J Addict. 2020;29(4):340-344. 10.1111/ajad.13027 32223045 10.1111/ajad.13027

[5] **Reid MC, Guo Z, Towle VR, Kerns RD, Concato J.** Pain-related disability among older male veterans receiving primary care. J Gerontol A Biol Sci Med Sci. 2002;57(11):M727-732. 10.1093/gerona/57.11.m727 12403801 10.1093/gerona/57.11.m727

[6] **Krebs EE, Gravely A, Nugent S, et al.** Effect of opioid vs nonopioid medications on pain-related function in patients with chronic back pain or hip or knee osteoarthritis pain: The SPACE randomized clinical trial. JAMA. 2018;319(9):872-882. 10.1001/jama.2018.0899 29509867 10.1001/jama.2018.0899 PMC5885909

[7] **Kuehn BM.** AHRQ: Little evidence for opioids in managing long-term chronic pain. JAMA. 2014;312(12):1185. 10.1001/jama.2014.12752

[8] **Manchikanti L, Kaye AM, Kaye, Alan D.** Current state of opioid therapy and abuse. Curr Pain Headache Rep. 2016;20(34). 10.1007/s11916-016-0564-x 10.1007/s11916-016-0564-x 27048483

[9] **Sjøgren P, Grønbæk M, Peuckmann V, Ekholm O.** A population-based cohort study on chronic pain: The role of opioids. Clin J Pain. 2010;26(9):763-769. 10.1097/AJP.0b013e3181f15daf 20842015 10.1097/AJP.0b013e3181f15daf

[10] **Eriksen J, Sjøgren P, Bruera E, Ekholm O, Rasmussen NK.** Critical issues on opioids in chronic non-cancer pain: An epidemiological study. Pain. 2006;125(1–2):172-179. 10.1016/j.pain.2006.06.009 16842922 10.1016/j.pain.2006.06.009

[11] Interagency Pain Research Coordinating Committee. National Pain Strategy: A Comprehensive Population Health-Level Strategy for Pain. National Academies Press; 2017. Accessed July 1, 2024. https://www.iprcc.nih.gov/sites/default/files/documents/NationalPainStrategy_508C.pdf

[12] **Krebs EE, Becker WC, Nelson DB, et al.** Care models to improve pain and reduce opioids among patients prescribed long-term opioid therapy: The VOICE randomized clinical trial. JAMA Intern Med. Published online December 9, 2024. 10.1001/jamainternmed.2024.6683 10.1001/jamainternmed.2024.6683 PMC11791716 39652356

[13] **Henry SG, Bell RA, Fenton JJ, Kravitz RL.** Communication about chronic pain and opioids in primary care: impact on patient and physician visit experience. Pain. 2018;159(2):371-379. 10.1097/j.pain.0000000000001098 29112009 10.1097/j.pain.0000000000001098 PMC5934342

[14] **Matthias MS, Parpart AL, Nyland KA, et al.** The patient–provider relationship in chronic pain care: Providers’ perspectives. Pain Med. 2010;11(11):1688-1697. 10.1111/j.1526-4637.2010.00980.x 21044259 10.1111/j.1526-4637.2010.00980.x

[15] **Matthias MS, Krebs EE, Collins LA, Bergman AA, Coffing J, Bair MJ.** “I’m not abusing or anything”: Patient-physician communication about opioid treatment in chronic pain. Patient Educ Couns. 2013;93(2):197-202. 10.1016/j.pec.2013.06.021 23916677 10.1016/j.pec.2013.06.021

[16] **Frantsve LME, Kerns RD.** Patient–provider interactions in the management of chronic pain: Current findings within the context of shared medical decision making. Pain Med. 2007;8(1):25-35. 10.1111/j.1526-4637.2007.00250.x 17244101 10.1111/j.1526-4637.2007.00250.x

[17] **Kenny DT.** Constructions of chronic pain in doctor–patient relationships: Bridging the communication chasm. Patient Educ Couns. 2004;52(3):297-305. 10.1016/S0738-3991(03)00105-8 14998600 10.1016/S0738-3991(03)00105-8

[18] **Matthias MS, Krebs EE, Bergman AA, Coffing JM, Bair MJ.** Communicating about opioids for chronic pain: A qualitative study of patient attributions and the influence of the patient–physician relationship. Eur J Pain. 2014;18(6):835-843. 10.1002/j.1532-2149.2013.00426.x 24921073 10.1002/j.1532-2149.2013.00426.x

[19] **Upshur CC, Bacigalupe G, Luckmann R.** “They don’t want anything to do with you”: Patient views of primary care management of chronic pain. Pain Med. 2010;11(12):1791-1798. 10.1111/j.1526-4637.2010.00960.x 21029353 10.1111/j.1526-4637.2010.00960.x

[20] **Jonsdottir T, Gunnarsdottir S, Oskarsson GK, Jonsdottir H.** Patients’ perception of chronic-pain-related patient–provider communication in relation to sociodemographic and pain-related variables: A cross-sectional nationwide study. Pain Manag Nurs. 2016;17(5):322-332. 10.1016/j.pmn.2016.07.001 27553131 10.1016/j.pmn.2016.07.001

[21] **Werner A, Malterud K.** It is hard work behaving as a credible patient: Encounters between women with chronic pain and their doctors. Soc Sci Med. 2003;57(8):1409-1419. 10.1016/S0277-9536(02)00520-8 12927471 10.1016/s0277-9536(02)00520-8

[22] **Esquibel AY, Borkan J.** Doctors and patients in pain: Conflict and collaboration in opioid prescription in primary care. Pain. 2014;155(12):2575-2582. 10.1016/j.pain.2014.09.018 25261714 10.1016/j.pain.2014.09.018

[23] **David Michaelson & Company, LLC.** Voices of Chronic Pain: A National Study Conducted for American Pain Foundation. American Pain Foundation; 2006. http://www.painfoundation.org/ Voices/VoicesSurveyReport.pdf

[24] **McCarberg BH, Nicholson BD, Todd KH, Palmer T, Penles L.** The impact of pain on quality of life and the unmet needs of pain management: Results from pain sufferers and physicians participating in an Internet survey. Am J Ther. 2008;15(4):312-320. 10.1097/MJT.0b013e31818164f2 18645331 10.1097/MJT.0b013e31818164f2

[25] **Dueñas M, Ojeda B, Salazar A, Mico JA, Failde I.** A review of chronic pain impact on patients, their social environment and the health care system. J Pain Res. 2016;9:457-467. 10.2147/JPR.S105892 27418853 10.2147/JPR.S105892 PMC4935027

[26] **Kroenke K, Krebs EE, Wu J, Yu Z, Chumbler NR, Bair MJ.** Telecare collaborative management of chronic pain in primary care: a randomized clinical trial. JAMA. 2014;312(3):240-248. 10.1001/jama.2014.7689 25027139 10.1001/jama.2014.7689

[27] **Seal K, Becker W, Tighe J, Li Y, Rife T.** Managing chronic pain in primary care: It really does take a village. J Gen Intern Med. Published online March 23, 2017. 10.1007/s11606-017-4047-5 10.1007/s11606-017-4047-5 PMC5515788 28337689

[28] **Gibson CJ, Grasso J, Li Y, et al.** An integrated pain team model: Impact on pain-related outcomes and opioid misuse in patients with chronic pain. Pain Med. Published online February 25, 2020. 10.1093/pm/pnaa003 10.1093/pm/pnaa003 32100002

[29] **Sellinger JJ, Gilstad-Hayden K, Lazar C, et al.** Impact of the COVID-19 pandemic on participants in pragmatic clinical trials for chronic pain: Implications for trial outcomes and beyond. Pain Med. 2024;25(Supplement_1):S17-S27. 10.1093/pm/pnae060 10.1093/pm/pnae060 PMC11548862 39514885

[30] **Griffith KN, Asfaw DA, Childers RG, Wilper AP.** Changes in US veterans’ access to specialty care during the COVID-19 pandemic. JAMA Netw Open. 2022;5(9):e2232515. 10.1001/jamanetworkopen.2022.32515 36125814 10.1001/jamanetworkopen.2022.32515 PMC9490495

[31] **Guest G, Bunce A, Johnson L.** How many interviews are enough?: An experiment with data saturation and variability. Field Methods. 2006;18(1):59-82. 10.1177/1525822X05279903

[32] **Purcell N, Becker WC, Zamora KA, et al.** Tailored to fit: How an implementation framework can support pragmatic pain care trial adaptation for diverse Veterans Affairs clinical settings. Med Care. 2020;58 Suppl 2 9S:S80-S87. 10.1097/MLR.0000000000001376 10.1097/MLR.0000000000001376 PMC7444583 32826776

[33] **Hagedorn HJ, Wisdom JP, Gerould H, et al.** Alcohol use disorder pharmacotherapy and treatment in primary care (ADaPT-PC) trial: Impact on identified barriers to implementation. Subst Abuse. 2022;43(1):1043-1050. 10.1080/08897077.2022.2060444 10.1080/08897077.2022.2060444 35467489

[34] **Hagedorn HJ, Wisdom JP, Gerould H, et al.** Implementing alcohol use disorder pharmacotherapy in primary care settings: a qualitative analysis of provider-identified barriers and impact on implementation outcomes. Addict Sci Clin Pract. 2019;14(1):24. 10.1186/s13722-019-0151-7 31291996 10.1186/s13722-019-0151-7 PMC6617941

[35] **Hagedorn HJ, Stetler CB, Bangerter A, Noorbaloochi S, Stitzer ML, Kivlahan D.** An implementation-focused process evaluation of an incentive intervention effectiveness trial in substance use disorders clinics at two Veterans Health Administration medical centers. Addict Sci Clin Pract. 2014;9(1):12. 10.1186/1940-0640-9-12 25008457 10.1186/1940-0640-9-12 PMC4106217

[36] **Purcell N, Zamora K, Tighe J, Li Y, Douraghi M, Seal K.** The Integrated Pain Team: A mixed-methods evaluation of the impact of an embedded interdisciplinary pain care intervention on primary care team satisfaction, confidence, and perceptions of care effectiveness. Pain Med Malden Mass. 2018;19(9):1748-1763. 10.1093/pm/pnx254 10.1093/pm/pnx254 29040715

[37] **Purcell N, Zamora K, Gibson C, et al.** Patient experiences with integrated pain care: A qualitative evaluation of one VA’s biopsychosocial approach to chronic pain treatment and opioid safety. Glob Adv Health Med. 2019;8:2164956119838845. 10.1177/2164956119838845 31041143 10.1177/2164956119838845 PMC6480990

[38] **Purcell N.** Matrix-based qualitative analysis. Presented at: Qualitative & Mixed Methods Research Course, Implementation Science Program, Department of Epidemiology and Biostatistics; February 2021; University of California, San Francisco.

[39] **Hamilton AB.** Qualitative methods in rapid turn-around health services research. Presented at: Health Services Research & Development Cyberseminar; December 11, 2013. Accessed June 3, 2016. http://www.hsrd.research.va.gov/for_researchers/cyber_seminars/archives/video_archive.cfm?SessionID=780

[40] **Purcell N, Zamora K, Bertenthal D, Abadjian L, Tighe J, Seal KH.** How VA Whole Health Coaching can impact veterans’ health and quality of life: A mixed-methods pilot program evaluation. Glob Adv Health Med. 2021;10:2164956121998283. 10.1177/2164956121998283 33747639 10.1177/2164956121998283 PMC7940726

[41] **Purcell N, Zamora K, Tighe J, Li Y, Douraghi M, Seal K.** The Integrated Pain Team: A mixed-methods evaluation of the impact of an embedded interdisciplinary pain care intervention on primary care team satisfaction, confidence, and perceptions of care effectiveness. Pain Med. Published online October 12, 2017. 10.1093/pm/pnx254 10.1093/pm/pnx254 29040715

[42] **Haley SJ, Pinsker EA, Gerould H, Wisdom JP, Hagedorn HJ.** Patient perspectives on alcohol use disorder pharmacotherapy and integration of treatment into primary care settings. Subst Abuse. 2019;40(4):501-509. 10.1080/08897077.2019.1576089 10.1080/08897077.2019.1576089 30829127

[43] **Birks M, Chapman Y, Francis K.** Memoing in qualitative research: Probing data and processes. J Res Nurs. 2008;13(1):68-75. 10.1177/1744987107081254

[44] **Hamilton AB.** Rapid qualitative analysis: Updates/developments. Presented at: VA Health Services Research & Development Cyberseminar; September 29, 2020. Accessed November 5, 2020. https://www.hsrd.research.va.gov/for_researchers/cyber_seminars/archives/video_archive.cfm?SessionID=3846

[45] **Abraham TH, VanTiem J.** Using qualitative summary templates and matrix displays to assess factors that impact the pace of implementation. Presented at: VA Health Services Research & Development Cyberseminar; June 6, 2021. Accessed August 5, 2021. https://www.hsrd.research.va.gov/for_researchers/cyber_seminars/archives/video_archive.cfm?SessionID=3996

[46] **Palinkas LA, Mendon SJ, Hamilton AB.** Innovations in mixed methods evaluations. Annu Rev Public Health. 2019;40:423-442. 10.1146/annurev-publhealth-040218-044215 30633710 10.1146/annurev-publhealth-040218-044215 PMC6501787

[47] **Vindrola-Padros C, Johnson GA.** Rapid techniques in qualitative research: A critical review of the literature. Qual Health Res. Published online July 15, 2020. 10.1177/1049732320921835 10.1177/1049732320921835 32667277

[48] **Taylor B, Henshall C, Kenyon S, Litchfield I, Greenfield S.** Can rapid approaches to qualitative analysis deliver timely, valid findings to clinical leaders? A mixed methods study comparing rapid and thematic analysis. BMJ Open. 2018;8(10):e019993. 10.1136/bmjopen-2017-019993 30297341 10.1136/bmjopen-2017-019993 PMC6194404

[49] **Kowalski CP, Nevedal AL, Finley EP, et al.** Planning for and Assessing Rigor in Rapid Qualitative Analysis (PARRQA): a consensus-based framework for designing, conducting, and reporting. Implement Sci. 2024;19(1):71. 10.1186/s13012-024-01397-1 39394597 10.1186/s13012-024-01397-1 PMC11468362

[50] **Hsieh HF, Shannon SE.** Three approaches to qualitative content analysis. Qual Health Res. 2005;15(9):1277-1288. 10.1177/1049732305276687 16204405 10.1177/1049732305276687

[51] **Zulman DM, Haverfield MC, Shaw JG, et al.** Practices to foster physician presence and connection with patients in the clinical encounter. JAMA. 2020;323(1):70-81. 10.1001/jama.2019.19003 31910284 10.1001/jama.2019.19003

[52] **Kim K, Rendon I, Starkweather A.** Patient and provider perspectives on patient-centered chronic pain management. Pain Manag Nurs Off J Am Soc Pain Manag Nurses. 2021;22(4):470-477. 10.1016/j.pmn.2021.02.003 10.1016/j.pmn.2021.02.003 33744105

[53] **Matthias MS, Bolla AL, Bair SM, et al.** Communication and Activation in Pain to Enhance Relationships and Treat Pain with Equity (COOPERATE): A qualitative analysis of a tailored coaching program for black patients with chronic pain. J Gen Intern Med. 2024;39(2):222-228. 10.1007/s11606-023-08410-1 37726645 10.1007/s11606-023-08410-1 PMC10853119

